# René Dubos, Friend of the Good Earth: Microbiologist, Medical Scientist, Environmentalist

**DOI:** 10.3201/eid1205.060354

**Published:** 2006-05

**Authors:** Russell Schaedler

**Affiliations:** * Jefferson Medical College of Thomas Jefferson University, Philadelphia, Pennsylvania, USA

**Keywords:** tuberculosis, tubercle bacilli, microbiology

René Dubos: Friend of the Good Earth is the only biography that documents Dubos's early life and progression into one of the 20th century's great scientist-philosophers. It is well researched and adequately referenced, when references are available. This book will be an invaluable resource, with some caveats.

This book suggests that philosophy came first and science followed. In reality, The White Plague, Mirage of Health, Torch of Life, Man Adapting, and So Human an Animal were written after the seminal scientific work had been done or while it was in progress. In science, Dubos never made exaggerated claims or overstepped scientific evidence. In his books and lectures, however, he could expand his thoughts, ideas, and hunches without recrimination.

The years of Dubos's scientific career after his early success culturing *Mycobacterium tuberculosis* in dispersed culture and the less-than-successful attempts to develop diagnostic tests are given little attention. Many years of work on acid-fast organisms did little to expand knowledge of tubercle bacilli, but the work opened avenues of research on the host's responses to infection. The use of living and killed bacillus Calmette-Guérin and endotoxin aided our understanding of the multiplicity of immune responses.

Moberg states that Walsh McDermott and the Cornell group grew 50 L of tubercle bacilli every day; while harvesting the organisms, one of the investigators became ill. This statement is wrong in 2 respects. First, to grow 50 L per day would take a pilot plant, and second, the work was done in Dubos's laboratory. Every week in Dubos's laboratory, 50 L media was inoculated so a mass of tubercle bacilli surface growth could be obtained. Four weeks later, the 50 L surface culture was harvested by using a number 2 centrifuge fitted with a basket filter. Opening the centrifuge aerosolized killed bacilli. After several exposures, a colleague had a severe generalized sensitivity reaction. Then the tubercle bacilli hit the fan! The old centrifuge was put into a specially constructed wooden box (limed oak, to match the rest of the laboratory cabinets), with a large, gasketed door and rubber gauntlet gloves to allow manipulation of the centrifuge, and the entire cabinet was vented to the roof. No one became ill again.

In another minor error, a facility known as Mousehatten House was designed to easily feed and care for large numbers of animals on special diets, so that their gut flora could be evaluated. Each animal would have its own unit with food, drinking fountain, and waste collection. Plans for the construction had to be submitted to New York City licensing and inspection authorities because it was a new animal unit in a new laboratory. Unfortunately, the plans were returned as inadequate—no provision for fire escapes. Of course, Dubos thought this story was hilarious, and all of us recounted it at various gatherings. After the story appeared in the New Yorker, I found the front portion of an old Christmas card depicting a toy train on my desk. On the back Dubos had written, "'When we are no longer children we are already dead'—Brancusi" and "'Genius is childhood recaptured'—Baudelaire."

The creation of a specific-pathogen-free mouse by Nelson and Collins gave the Dubos group a new and reliable standardized "fuzzy test tube." These animals provided the impetus for work on gut flora, the association of microbes with mucosal surfaces, and some of the earliest work with probiotics. These animals were also used in Dubos's students' social science and crowding experiments. It was a time of scientific advances and the foundation for much of Dubos's work in philosophy. This decade of Dubos's scientific work is glossed over in a cursory manor. The author even quotes one of Dubos's colleagues as stating that the work isn't worth a "hill of beans."

Also, some of Dubos's great talents are not mentioned. He did not like statistics and avoided using them by repeating his experiments over and over, deleting parameters that seemed fruitless and adding others that seemed promising. This repetition gave his experiments numbers that were large enough not to need statistical analysis, and none of his published articles ever had to be retracted. His conclusions were based on the data at hand; he never overstepped this boundary. Dubos also had the ability to analyze raw data, focusing on important aspects and suggesting new and fruitful experiments.

This book often mentions of Dubos's farm in Garrison, New York. Planting trees and hunting for water were among his prime pleasures. Behind his house was a beautiful grotto in which grew a lovely hepatica. His neighbor, an elderly European farmer, told him, "There's a spring here. Drill through that rock and you will find water." Dubos began to drill with diligent, laborious work, and after ≈6 months, he had drilled a 1.5-inch hole, 30 inches deep into the rock without finding water. During all these hours of labor, Dubos was always thinking about experiments, contemplating what he would do that week in the laboratory, or honing his thoughts on a new lecture or book, so the time was not wasted. The neighbor shared Dubos's disappointment in not finding water and obtained a stick of dynamite to put into the hole. The dynamite obliterated the rock, the beautiful grotto, and the lovely hepatica, but no spring was ever found.

Dubos enjoyed wildflowers and was an expert on the identification of wildflowers of New York. He had a looseleaf portfolio of the wildflowers of New York by the University of New York State Museum from 1921. The illustrations were lithographs of color drawings. He cut them apart and took those he had not yet seen on his walks and excursions. I dare say he saw and identified most, if not all, of the flowers in that portfolio. He was proud of this accomplishment and was delighted when the occasional guest to his farm noticed or knew of the wildflowers. (Most of his scientific colleagues were not in this league). His passion for planting trees was great, but he did not have the strength to dig deep holes in that rocky landscape, and watering in many places was difficult. However, as in all his endeavors, he persisted, replanting new trees where previous plantings failed to survive. Most plantings were not done according to a grand plan but of necessity, following the curvature of the driveway and places where he could dig. Hemlocks and dogwoods were the species of choice.

Dubos had difficulty with some personal relationships. If he knew a particular person he did not like was in town, he would hide in his office or seclude himself in his apartment. This inability to confront a person extended to colleagues and visitors, but others were always welcome. He kept a nervous distance from people of authority. Whenever he had to report to his superiors or go to Washington to testify or present to a committee, the telltale signs appeared several days in advance. His lips became coated with a white film from constantly chewing Gelusil, and herpetic lesions appeared on his lip. These signs indicated he had to perform one of his distasteful duties.

A favorite book of Dubos's was The Unseen World, a result of the first Christmas Lectures at Rockefeller University. In this whimsical book, he gained a rapport with his audience, and they received a different understanding of life. Dubos wrote, "this microbiology as a way of life, fortunately not incompatible with more earthy ways." As one of the great scientists of the 20th century, Dubos in his later decades turned to philosophy to better spread his views on humans and their reaction to all things around them. We should remember that throughout his life, Dubos was "so human an animal."

